# *Haloferax volcanii*, a Prokaryotic Species that Does Not Use the Shine Dalgarno Mechanism for Translation Initiation at 5′-UTRs

**DOI:** 10.1371/journal.pone.0094979

**Published:** 2014-04-14

**Authors:** Piet Kramer, Katrin Gäbel, Friedhelm Pfeiffer, Jörg Soppa

**Affiliations:** 1 Institute for Molecular Biosciences, Biocentre, Goethe-University, Frankfurt, Germany; 2 Max-Planck-Institute for Biochemistry, Martinsried, Germany; University of Florida, United States of America

## Abstract

It was long assumed that translation initiation in prokaryotes generally occurs via the so-called Shine Dalgarno (SD) mechanism. Recently, it became clear that translation initiation in prokaryotes is more heterogeneous. In the haloarchaeon *Haloferax volcanii,* the majority of transcripts is leaderless and most transcripts with a 5′-UTR lack a SD motif. Nevertheless, a bioinformatic analysis predicted that 20–30% of all genes are preceded by a SD motif in haloarchaea. To analyze the importance of the SD mechanism for translation initiation in haloarchaea experimentally the monocistronic *sod* gene was chosen, which contains a 5′-UTR with an extensive SD motif of seven nucleotides and a length of 19 nt, the average length of 5′UTRs in this organism. A translational fusion of part of the *sod* gene with the *dhfr* reporter gene was constructed. A mutant series was generated that matched the SD motif from zero to eight positions, respectively. Surprisingly, there was no correlation between the base pairing ability between transcripts and 16S rRNA and translational efficiency *in vivo* under several different growth conditions. Furthermore, complete replacement of the SD motif by three unrelated sequences did not reduce translational efficiency. The results indicate that *H. volcanii* does not make use of the SD mechanism for translation initiation in 5′-UTRs. A genome analysis revealed that while the number of SD motifs in 5′-UTRs is rare, their fraction within open reading frames is high. Possible biological functions for intragenic SD motifs are discussed, including re-initiation of translation at distal genes in operons.

## Introduction

Translation is a very important step in the expression of genetic information in all three domains of life. It can be subdivided into initiation, elongation, termination and ribosome recycling. Translation initiation is the rate-limiting step, and therefore the regulation of translation typically occurs during the initiation phase. Several decades ago the so-called Shine Dalgarno mechanism for translation initiation was discovered, and it was long thought to be the default mechanism in all prokaryotes [1], [2]. It is operating on transcripts with 5′-UTRs that contain the so called SD motif of 4–8 nt a few nucleotides upstream of the start codon. The function of this mechanism is based on the base-pairing of the SD motif with the anti SD sequence (aSD) located at the 3′-end of the 16S rRNA. The optimal spacing of the SD sequence to the start codon was determined to be 5+/−2 nt for *E. coli* [3]. The direct interaction of the SD/aSD duplex enables the existence of polycistronic transcripts that are comprised of several open reading frames (ORFs), which have their own initiation signals and are thus able to attract cytoplasmic ribosomes at all distal genes.

*In vitro* it has been shown that the affinity of transcripts with a SD motif to the 30S ribosomal subunit is more than an order of magnitude higher than that of transcripts lacking an SD motif [4]. However, the influence on the amount of protein produced *in vitro* was limited at low transcript concentrations and non-existent at high transcript concentrations, leading to a model that translational efficiency is kinetically controlled at steps following the SD/aSD interaction [4]. In stark contrast, a plethora of experimental studies have been performed with *E. coli* showing that the SD motif is very important for translational efficiency *in vivo*, and only very few examples will be mentioned. A point mutation of the SD sequence led to a severe inhibition of translation initiation by as much as 97% [5], while a complete mutation of the SD sequence led to a total inhibition of translation [6]. A single point mutation in the anti-SD motif at the 3′-end of the 16S rRNA resulted in a severe reduction of the production of many proteins, underscoring the importance of the SD/aSD interaction for the majority of *E. coli* genes [7]. The localization of a SD motif between two AUG start codons led to a more than 60-fold increased protein production from the downstream start codon [8]. The expression of transcripts with a row of SD-like motifs led to a depletion of free 30S ribosomal subunits and a severe inhibition of growth [9]. A very elegant proof that the SD/aSD interaction is essential for translation initiation was the generation of a “specialized ribosome system” (or orthogonal ribosome system). The 3′-end of the 16S rRNA was mutated from the aSD motif to the SD motif, and the resulting “specialized ribosomes” exclusively translated transcripts that contained the aSD motif instead of the SD motif upstream of the start codon [6]. In addition, the structure of the ribosome together with a SD motif has been solved [10], [11]. Several reviews summarize the importance of the SD motif for translation initiation in *E. coli* [12]–[14].

However, in recent years it became evident that translation initiation in prokaryotes might be more complex, that additional translation initiation mechanisms exist, and that the results obtained with *E. coli* cannot be extrapolated to all bacteria or all prokaryotes [15]–[17]. An extensive analysis of 141 bacterial and 21 archaeal genomes revealed a high divergence of the occurrences of SD motifs. The fractions of genes preceded by a SD motif ranged from 11% to more than 90% in different phylogenetic groups of prokaryotes, and were very similar within phylogenetic groups [18]. A subsequent bioinformatic analysis of 277 prokaryotic genomes used a “SD index” and came to the same conclusion. It was postulated that the SD motif might have been lost several times independently in evolution in different phylogenetic phyla [16]. Bacterial phyla with extremely low SD indices were for example Bacteroidetes (-0.149) and cyanobacteria (0.012), while the indices were very high for Firmicutes (0.636) or Fusobacteria (0.607). There are two different types of transcripts that lack a SD motif, i.e. leaderless transcripts devoid of a 5′-UTR and transcripts with a 5′-UTR lacking a SD motif.

Initiation on leaderless transcript is thought to be the evolutionary oldest mechanism, because leaderless mRNAs are efficiently translated in all three domains of life [19], [20]. In some archaeal species it is the predominant mechanism [21]–[24], and in lower eukaryotes like *Giardia lamblia* all transcripts are leaderless [25], [26]. A bioinformatic analysis of 953 bacterial genomes has revealed that leaderless transcripts are also widespread in many bacteria, but that they are not predominant [27]. In contrast to all other mechanisms initiation on leaderless transcripts requires the undissociated 70S/80S ribosome and does not start with the small ribosomal subunit [21], [23]. It also deviates from other mechanisms in its strict requirement for AUG as a start codon, while GUG and UUG are non-functional in leaderless initiation [28].

The molecular mechanism of translation initiation on transcripts with SD-less 5′-UTRs is less clear. In *E. coli* it is thought to involve the ribosomal protein S1, which can bind AU-rich regions in the 5′-UTR [16]. However, in several groups of bacteria it is unclear whether S1 also fulfills this function and S1 is totally lacking in Mollicutes and Archaea. Nevertheless, transcripts with SD-less 5′-UTRs were shown to be efficiently translated in haloarchaea *in vivo* [28]. The three start codons AUG, GUG and UUG are all functional for initiation. It could be excluded that the mechanism involves ribosome scanning from the 5′-end of the transcript. However, molecular details and the involvement of initiation factors are currently unknown [28].

While it is clear that the majority of transcripts in haloarchaea and several other groups of archaea are leaderless and that they contain, in addition, a large fraction of transcripts with SD-less 5′-UTRs, until now only two experimental studies have been performed that address the importance of the SD motif in archaea. An *in vitro* translation system has been established for *Sulfolobus solfataricus*, which was used to study different aspects of translation initiation [21], [29]–[32]. Notably, the substitution of two nucleotides of the SD motif resulted in a total inhibition of translation, indicating that the SD mechanism is operating in this crenarchaeal species [29].

Translation initiation of *gvpH*, the third gene in the *gvpFGHIJKLM* gas vesicle operon of *H. salinarum* has been studied in *H. volcanii in vivo* [33]. The upstream region, which is part of the *gvpG* open reading frame, was fused to a reporter gene. Thereby an artificial 5′-UTR was generated that in addition to the *gvp* sequences contained 10 nt of the *fdx* gene including the start codon. A linker scanning mutagenesis throughout this artificial 5′-UTR of the reporter transcript was performed and mutations in the region between -15 and -8 nt upstream of the start codon reduced translational efficiency. Therefore, it was concluded that the SD motif is important for translation initiation in haloarchaea. However, initiation at this internal region of a polycistronic mRNA was rather inefficient, and total removal of the region, resulting in a leaderless reporter transcript, led to a 14-fold increase in translational efficiency [33].

Because until now no native haloarchaeal 5′-UTR with a SD motif has been studied, we aimed at an experimental analysis of the importance of the SD motif for translation initiation in *H. volcanii in vivo*. A genome-wide bioinformatic analysis was performed and the *sod* gene encoding superoxide dismutase was detected as one of only few genes that contain an extended SD motif in its 5′-UTR. This motif is 8 nt long of which 7 nt match to the 3′ end of the 16S rRNA. A translational fusion of the *sod* 5′-UTR and the first 30 *sod* codons with the reporter gene *dhfr* was generated and a series of mutants was constructed that had base pairing capabilities with the 3′-end of the 16S rRNA ranging from zero to eight nucleotides. The steady state levels of the DHFR protein and the *dhfr* transcript were quantified, and translational efficiencies were calculated for *H. volcanii* cultures grown under different conditions. Furthermore, in an additional experiment the native SD motif of the *sod* transcript was replaced by three unrelated sequences and the effect on the translational efficiency was quantified. A bioinformatic analysis of the occurrence of extended SD motifs within open reading frames was performed. The results indicate that *H. volcanii* does not make use of the SD motif in 5′-UTRs for translation initiation, but that intragenic SD motifs are common. Putative functions of intragenic SD motifs are discussed.

## Results

### Bioinformatic analysis of the *H. volcanii* genome

**Figure 1:**
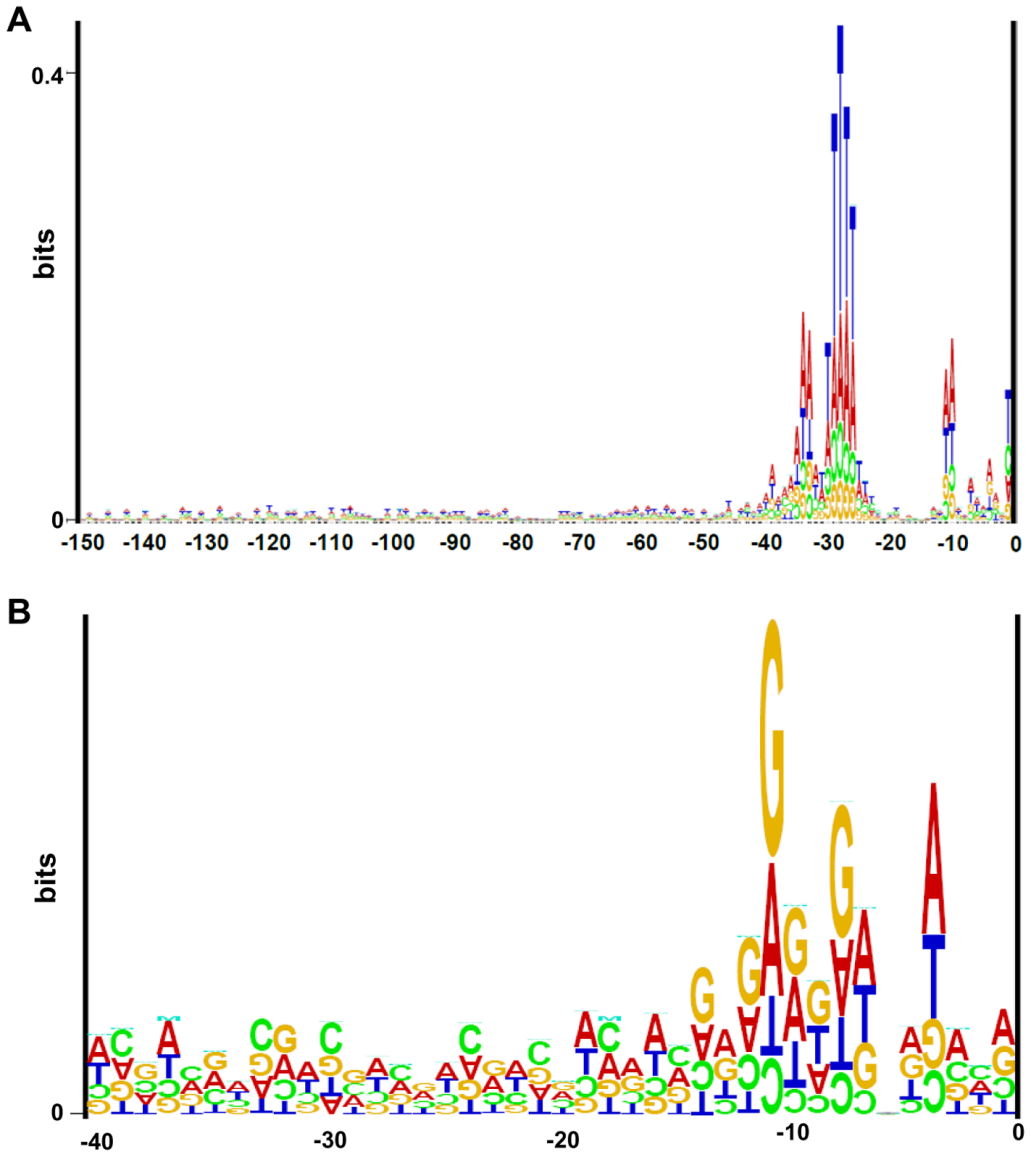
Bioinformatic analysis of the *H. volcanii* genome. A. Sequence logo of 5′ regions of 3025 putative monocistronic genes or proximal genes in operons with an intergenic distance at least 40 nt to the adjacent gene. B. Sequence logo of 5′ regions of 791 putative distal genes in operons with an intergenic distance of less than 10 nt to the adjacent gene.

A determination of 62 5′-ends of haloarchaeal transcripts indicated that the presence of SD motifs in 5′-UTRs might be rare in haloarchaea [24]. To get a more comprehensive overview, a bioinformatic analysis of the *H. volcanii* genome was performed. The sequences of all annotated protein coding genes from -150 to -1 nt relative to the initiation codon were extracted from the genome database “Halolex” [57]. The genes were sorted into four clusters. The first cluster was comprised of genes with a distance of at least 40 nt between their start codon and the upstream genes, because they were considered to be single genes or first genes in operons. This requirement is based on the assumption that 40 nt is the minimal length of an intergenic region that can harbor both a terminator of an upstream gene as well as a promoter of a downstream gene. This condition was fulfilled by 3025 genes, which is a high fraction of 76% of all genes of *H. volcanii*. A sequence Logo was generated to unravel sequence motifs with a fixed distance to the translational start sites (Fig. 1A). Four highly conserved motifs were retrieved, which are all known to be required for transcription initiation: 1) the BRE motif at −34/−33, 2) the TATA box at −29 to −25, 3) the WW motif around −10, and 4) a pyrimidine at position -1. The distances of the four transcription initiation motifs to the translational start codon revealed that transcription initiation and translation initiation at these genes coincide. Therefore, this genome-wide analysis underscores previous results obtained with a limited set of genes that typically transcripts in *H. volcanii* are leaderless. Notably, other motifs including a Shine Dalgarno motif were totally absent from this cluster of genes.

The second cluster of genes was comprised of genes that had the same direction of transcription and an intergenic distance of less than 10 nt to an upstream gene, which was taken as a strong indication that they were distal genes in operons. A sequence Logo of this cluster, which contains 791 genes (20% of all genes) is shown in Fig. 1B. A conserved motif was retrieved that resembled the SD motif GGAGGT and that had the optimal distance to the start codon of the downstream gene. However, the information content of the sequence Logo was very small (<0.3 bits), indicating that either the fraction of downstream genes that are preceded by an SD motif is not high or that the distance between the SD motif and the start codon is not well conserved (see below).

The third and the fourth cluster were comprised of genes that had the same direction of transcription and an intergenic distance of either 10 nt – 25 nt or 26 nt – 39 nt. The number of genes in these clusters were very small, together only 163 genes (4%). The sequence Logos of these clusters did not retrieve any conserved motifs with a fixed distance to the translational start site (data not shown).

The bioinformatic analysis presented above revealed that the presence of a SD motif in 5′-UTRs is not typical for *H. volcanii* transcripts. Nevertheless, a bioinformatic genome analysis had predicted that 20% of the genes of *H. salinarum* are preceded by a SD motif [18], and manual examination of the *H. volcanii* genome showed that several genes exist that are preceded by an extended SD motif in the optimal distance to the translational start site. For example, the *sod* gene (*HVO_2913*) encoding the superoxide dismutase had the extended SD motif GGAGGUUA with seven nucleotides (underlined) that could hybridize to the 3′-end of the 16S rRNA. The 5′-end of the *sod* transcript had been determined and the 5′-UTR was found to have a length of 19 nt [35], which is the average length of haloarchaeal 5′-UTRs [24]. Thus the SD motif encoded in the genome was indeed present in the *sod* transcript and could have a function in translation initiation. Northern blot analysis had revealed that the *sod* gene is transcribed into a monocistronic transcript [35]. These features made the *sod* gene an ideal example to study the importance of the SD motif in a native 5′-UTR of a haloarchaeal gene for translation initiation *in vivo* in its native host.

### Relevance of the SD/aSD complementarity for the translational efficiency *in vivo*

**Figure 2:**
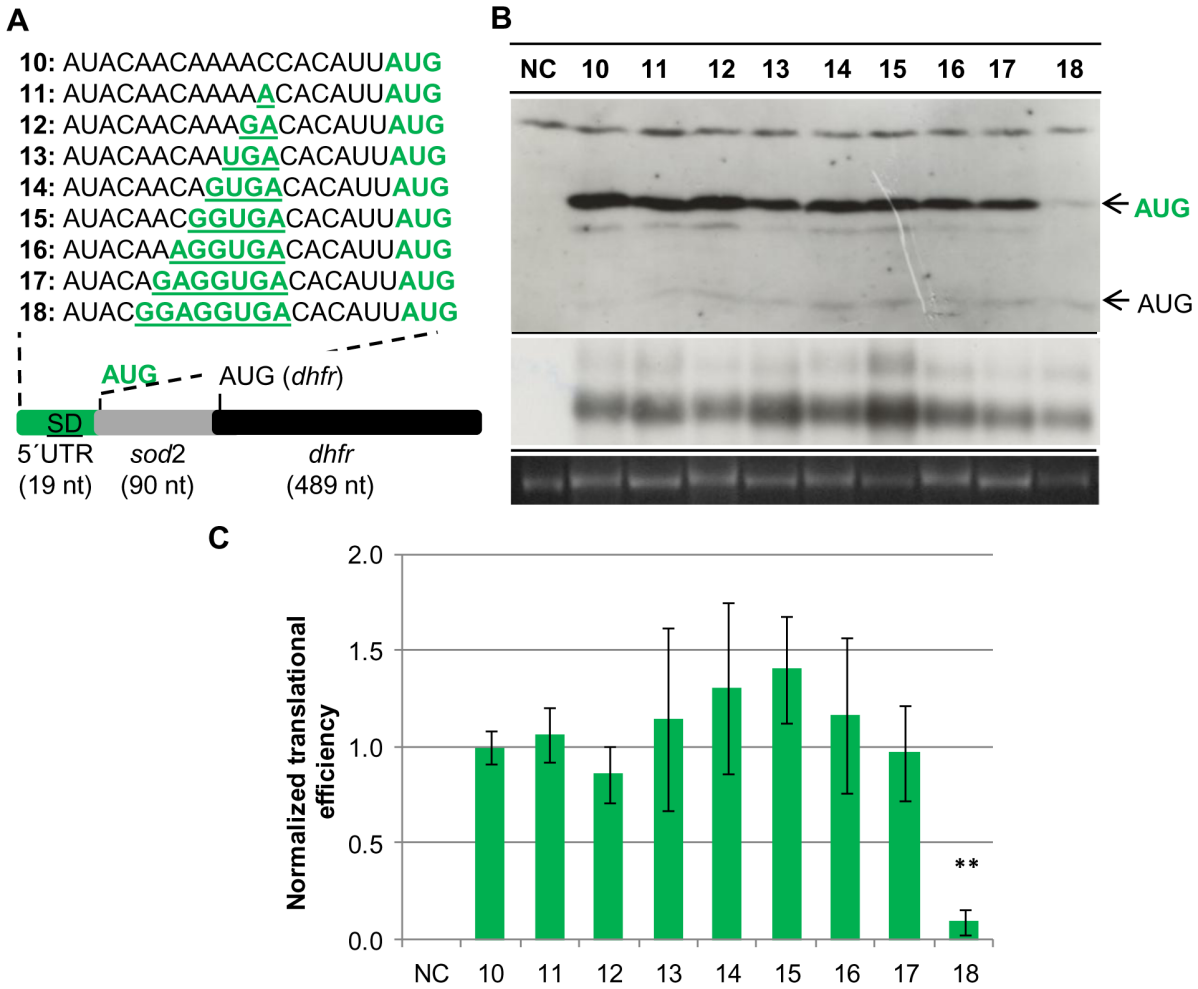
Translational efficiencies of a consecutive SD mutant series under standard conditions. For the comparative analysis of translational efficiencies H. volcanii cultures of pPK10-pPK18 and pNP10 negative control (without hdrA) were grown to mid-exponential growth phase under standard conditions (2.1 M NaCl, 42°C). A. Schematic overview of the reporter gene constructs pPK10-pPK18. The SD sequence (green and underlined) was mutated as indicated. B. One representative example of three independent experiments is shown for a Western blot analysis (upper panel), a Northern blot analysis (middle panel), and the 16S rRNA of an agarose gel used for normalization. The protein and transcript levels were analyzed as described in Experimental Procedures. The results are summarized in Table S2. C. Graphic representation of the normalized average translational efficiencies and their standard deviations (n = 3).

The *dhfr* reporter gene encoding dihydrofolate reductase was used successfully in *H. volcanii* to analyze transcription initiation, translation initiation, and translational regulation [28], [36], [37]. The DHFR protein level can be quantified by an enzymatic test as well as using a specific antibody, and the transcript level can be quantified by Northern blot analysis or qRT-PCR. A translational fusion of the 5′-UTR and the first 30 codons of the *sod* gene and the *dhfr* reporter gene was constructed (Fig. 2A). The first 30 *sod* codons were included to guarantee that translation initiation is only driven by features of the *sod* gene even if sequences downstream of the *sod* start codon would influence initiation efficiency. A series of nine mutants was constructed that had a base pairing potential to the 16S rRNA aSD sequence from zero to eight nt (Fig. 2A, green underlined). All mutant constructs were cloned into shuttle vector pSD1-R1/6 that enables propagation in *E. coli* as well as in *H. volcanii* [36]. All plasmids used in this study are summarized in Table S1. The *H. volcanii* strain H26Δ*dhfr* was transformed with the respective plasmids and grown in synthetic medium with glucose as carbon source to mid-exponential growth phase. Cells were harvested, the steady state levels of the DHFR protein level and the *dhfr* transcript were quantified, and translational efficiencies were calculated. The results of one representative experiment are shown in Fig. 2B. Average results and standard deviations of three independent biological replicates are summarized in Table S2, and normalized average translational efficiencies and their standard deviations are shown in Fig. 2C. There was no correlation between the degree of base-pairing potential of the respective mutants to the 16S rRNA and the translational efficiency for eight of the nine constructs. Transcripts with base pairing capabilities from zero to seven nucleotides to the 16S rRNA had virtually identical translational efficiencies.

The translational efficiency of plasmid pPK18 containing an extended SD motif of eight nucleotides was about tenfold reduced compared with the other eight transcripts. To exclude that this result might have been caused by mutations in the genome of this clone rather than from the variation of the SD motif, *H. volcanii* was newly transformed with the respective plasmid and the experiment was repeated using several independent clones. However, the results were the same, strongly indicating that the severe drop in translational efficiency in clone 18 compared to the other eight mutants is caused by the potential for a very strong interaction with the 16S aSD sequence.

**Figure 3:**
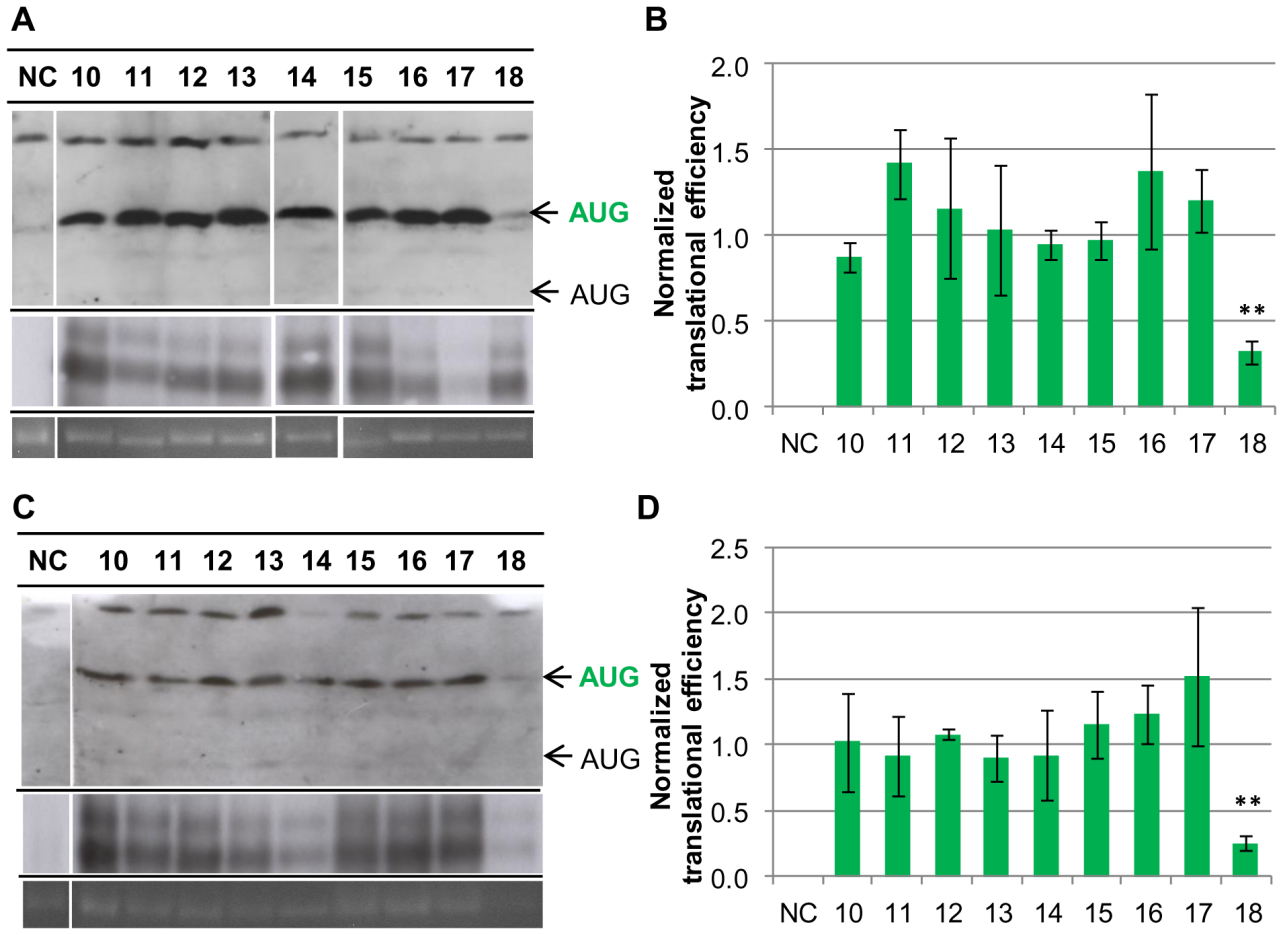
Translational efficiencies of the consecutive SD mutant series at reduced growth rates. For the comparative analysis of translational efficiencies *H. volcanii* cultures of pPK10-pPK18 and pNP10 negative control (NC) were grown to mid-exponential growth phase at the reduced temperature of 30°C (A, B) or with acetate as carbon and energy source (C, D). A. One representative example of three independent experiments is shown for a Western blot analysis (upper panel), a Northern blot analysis (middle panel), and the 16S rRNA of an agarose gel used for normalization. The protein and transcript levels were analyzed as described in Experimental Procedures. The results are summarized in Table S3. B. Graphic representation of the normalized average translational efficiencies and their standard deviations (n = 3). C. One representative example of three independent experiments is shown for a Western blot analysis (upper panel), a Northern blot analysis (middle panel), and the 16S rRNA of an agarose gel used for normalization. The protein and transcript levels were analyzed as described in Experimental Procedures. The results are summarized in Table S4. D. Graphic representation of the normalized average translational efficiencies and their standard deviations (n = 3).

To exclude the possibility that the SD motif might not be needed under optimal conditions, but might be crucial for translation initiation at low growth rates, translation efficiencies were also quantified in *H. volcanii* cultures grown at 30°C. At this temperature the growth rate is severely reduced. Again, cultures were grown to mid-exponential growth phase and the translational efficiencies of the nine constructs were quantified. The results of one representative experiment are shown in Fig. 3A, the average results of three independent biological replicates are shown in Fig. 3B, and the individual results of the three experiments are tabulated in Table S3. It is obvious that also under non-optimal conditions the translational efficiencies of eight of the nine mutants were identical. Again, translational efficiency of clone 18 was severely reduced, albeit not as much as under optimal conditions. Therefore, this data set confirmed the previous results and underscored that there is no correlation between the base pairing capability between the 5′-UTR of a transcript and the 16S rRNA aSD sequence from zero to seven base pairs. Furthermore, a base pairing capability of eight base pairs severely reduced translational efficiency.

To test a third condition, *H. volcanii* cultures were grown in synthetic medium with acetate as sole carbon source to mid-exponential growth phase. Acetate is a poor carbon source and results in a severely reduced growth rate. The results of one representative experiment are shown in Fig. 3C, average results of three replicates are shown in Fig. 3D, and the individual results of the three experiments are tabulated in Table S4. Again, transcripts with base pairing capabilities to the 16S rRNA from zero to seven nucleotides had virtually identical translational efficiencies, while the translational efficiency of the transcript with a 8 nt SD motif was much lower.

To test the significance of these results, one-way Anova tests were performed. As expected the small differences between the results of clones 10–17 were not significant, the p-values were 0.74 for growth on glucose, 0.51 for growth at 30°C, and 0.58 for growth on acetate. In contrast, clone 18 significantly differed from the other eight clones, the p-values were 0.0028 for growth on glucose, 0.0065 for growth at 30°C, and 0.0078 for growth on acetate. Taken together, the results strongly indicate that the SD mechanism for translation initiation is not operating on 5′-UTRs of haloarchaeal transcripts *in vivo* in cells growing with very different growth rates.

### Replacing the native sod SD motif by three unrelated sequences

**Figure 4:**
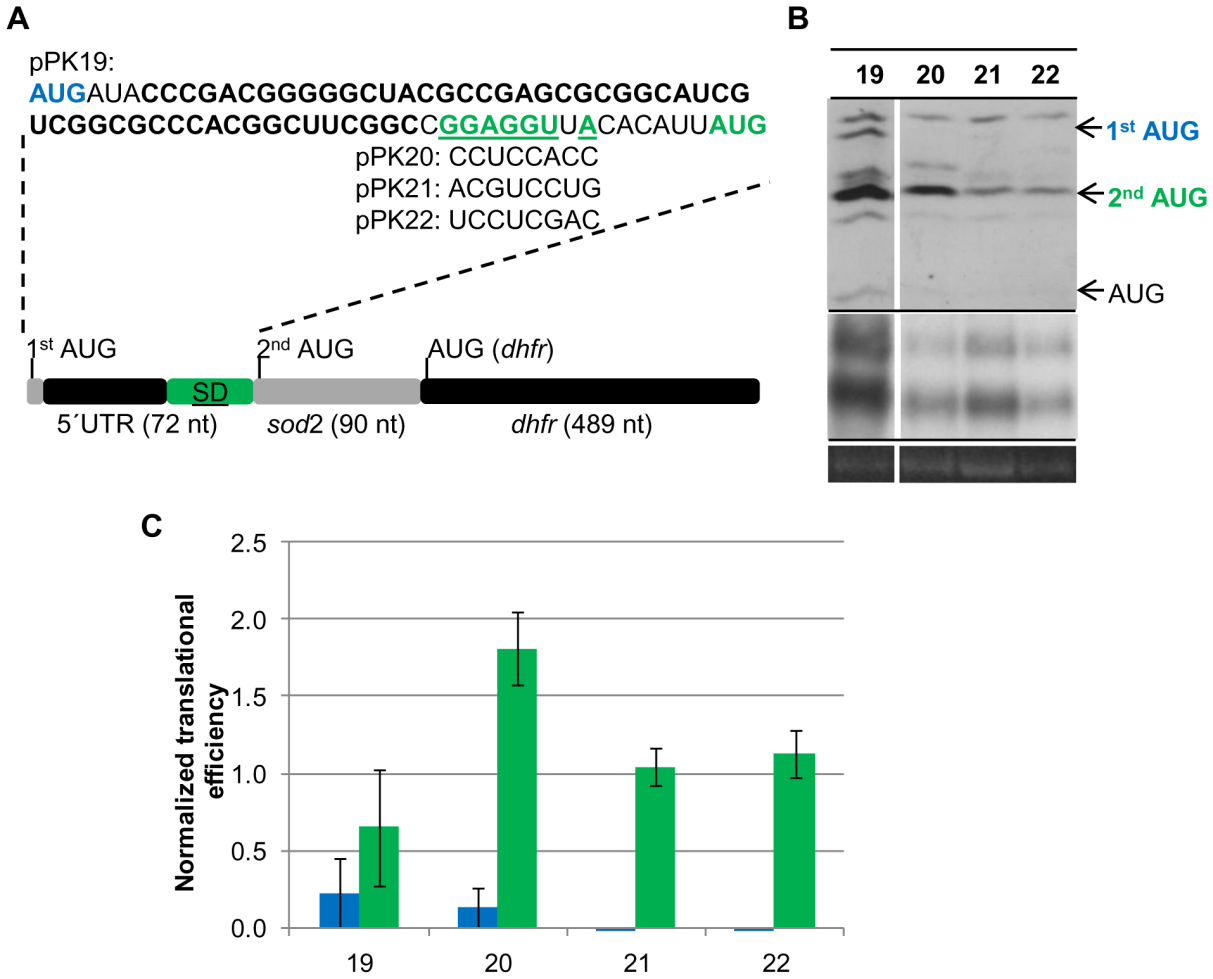
Translational efficiencies of different transcripts with and without an SD sequence. For the comparative analysis of translational efficiencies *H. volcanii* cultures of pPK19-pPK22 were grown to mid-exponential growth phase under standard conditions. A. Schematic overview of the reporter gene constructs pPK19-pPK22. The native SD motif of the *sod* gene (green and underlined) was replaced by three randomly generated unrelated sequences in the mutants pPK20-pPK22. A second leaderless translation start site AUG was introduced at the 5′-end of the 51 nt elongated *sod* 5′-UTR (blue). B. One representative example of three independent experiments is shown for a Western blot analysis (upper panel), a Northern blot analysis (middle panel), and the 16S rRNA of an agarose gel used for normalization. The protein and transcript levels were analyzed as described in Experimental Procedures. The results are summarized in Table S5. C. Graphic representation of the normalized average translational efficiencies and their standard deviations (n = 3). Results of proteins originating from the leaderless start codon are shown in blue, results of proteins originating from the *sod* start codon are shown in green.

The results described above revealed that reducing the base-pairing capability from seven to zero did not alter the translational efficiency. To broaden the significance of the results, three additional unrelated sequences with zero base-pairing capacity to the 16S rRNA were investigated. In this case a construct was generated that again contained a translational fusion of the first 30 codons of the *sod* gene with the *dhfr* reporter gene. However, the construct contained an extended 5′-UTR that was comprised of 15 nt of the *sod* 5′-UTR including the native SD motif and a 5′-extension with an in frame AUG start codon at the 5′-end (Fig. 4). This experimental design aimed at a direct comparison of the efficiencies of two different initiation mechanisms acting at the same transcript. The SD motif of the *sod* was replaced by three unrelated, randomly chosen sequences, which could not base-pair with the 16S rRNA. The results are summarized in Table S5 and are graphically shown in Fig. 4. In this construct with the extended SD motif of the *sod* gene leaderless translation initiation (shown in blue) was less than half as efficient as initiation at the native AUG of the *sod* (shown in green). Three additional bands were visible for clone 19: the band with the highest molecular weight was a cross-reaction with another protein and occurred in all clones, and two additional bands migth be degradation intermediates. The efficiency of leaderless translation initiation was even lower in clone 20 and so low that it could not be quantified in clones 21 and 22 (Fig. 4, upper arrow). This was unexpected, because leaderless transcripts are typically well-translated in haloarchaea. A consequence of the low translational efficiencies was that the proteins initiated at the two different start codons could not be quantified simultaneously using the same exposure time and thus the longer protein could not be used as an internal standard for the shorter protein, as originally planned. Replacement of the SD motif by unrelated sequences in all three cases did not result in a reduction of the translational efficiency, but the three clones 20–22 had higher translational efficiencies than the native *sod* 5′-UTR with the extended SD motif (Fig. 4, middle arrow). However, the translational efficiencies varied, for plasmids pPK21 and pPK22 it was about 50% higher than that of the control transcript with the extended SD motif of the *sod* gene, for plasmid pPK20 it was 2.5-fold higher than that of the control. These results showed that the region around -7 to -14 nt upstream of the start codon does effect translational efficiency in *H. volcanii*, albeit totally different than anticipated. Replacement of a SD motif of seven matching nucleotides with four unrelated sequences in one case did not influence initiation efficiency *in vivo*, and in three cases led to a significant enhancement of efficiency (Fig. 2, 3, 4).

### The occurrence of SD motifs within the open reading frames of genes

**Figure 5:**
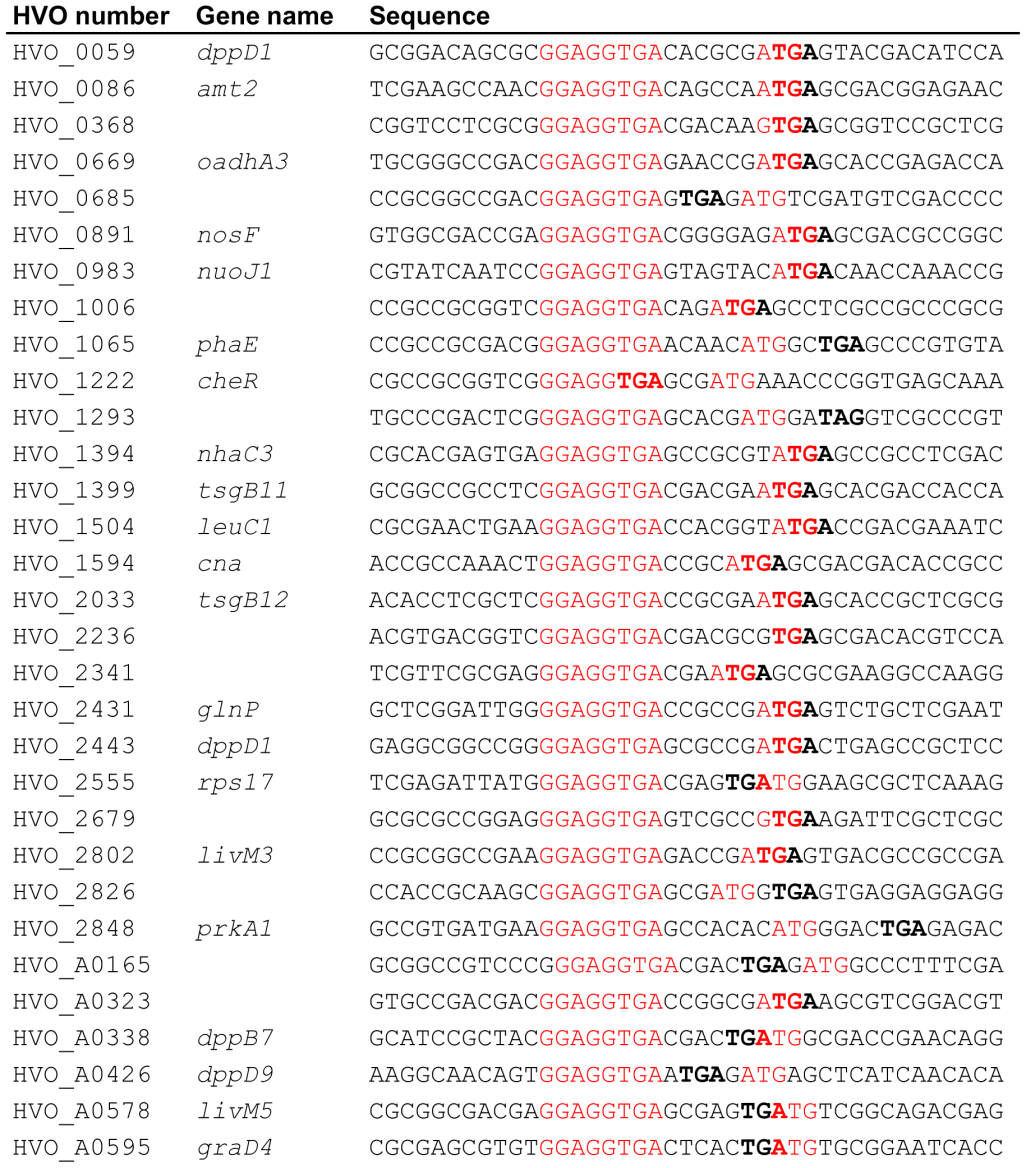
Spacing between the 8 nt SD motif to the start codon of the downstream gene. In 31 cases the extended 8 nt SD motif was found to be localized at the 3′-end near the stop codon. The sequences around the SD motif were retrieved from the genome sequence of *H. volcanii* and the gene identifier (HVO numbers) and sequences are shown. It turned out that all 31 genes were proximal genes in operons and were closely followed by a downstream gene. The SD motif and the start codon of the downstream gene are shown in red, and the stop codon of the upstream gene is shown in bold.

A genome-wide analysis of the localization of all ribosomes on all transcripts in *E. coli* and *Bacillus subtilis* had revealed that SD motifs exist also within open reading frames in these two species and that elongating ribosomes pause at these sites *in vivo* [38]. Introduction of an extended synthetic SD motif of eight nucleotides had severely reduced translational efficiency in *H. volcanii* (Fig. 2 and 3), possibly because the ribosomes were halted at initiation and switched to elongation only very slowly and inefficiently. To reveal whether *H. volcanii* might also make use of ribosomal pause sites during the elongation of translation both strands of the chromosome and the largest plasmid (comprising 3.5 Mbp of the total DNA content of 4 Mbp) were searched for the occurrence of the extended SD motif of eight nucleotides. The motif GGAGGTGA occurred 187 times, which is about 50% more frequent than the statistical expectation for a genome with a GC content of 66% and equal fractions of G/C and A/T on both strands. Only 17 of the 187 sites were in intergenic regions. Only four of these intergenic motifs had a distance of 5 +/− 2 nt to the start codon of the downstream gene and could be functional in translation initiation. The real number might even be smaller than four if one or several of the respective transcripts would be leaderless, as are the majority of haloarchaeal transcripts. Therefore, the extended 8 nt SD motif does only very seldom occur in 5′-UTRs of *H. volcanii*, if at all. This is in excellent agreement with the more than tenfold reduction in translational efficiency following the introduction of this motif in the 5′-UTR of the *sod* gene (Fig. 2 and 3). The motif occurred 78 times in antisense orientation within coding regions, and thus these sites could not be functional as pause sites. However, 90 of the extended SD motifs were localized in the sense orientation within open reading frames and thus could be pause signals for translating ribosomes. The majority of 59 SD motifs was dispersed throughout the ORFs, but a subclass of 31 of the extended 8 nt SD motifs were localized very close to the stop codon. All 31 genes of the latter subclass were parts of operons and were closely followed by a downstream gene. Fig. 5 shows the 31 sequences with highlighted SD motifs, stop codon of the respective ORF and start codon of the downstream ORF. All 31 extended SD motifs near the end of genes are in the ideal distance of 5 +/−2 nt to the start codon of the downstream gene and thus might function in translation re-initiation (the average distance is 5.55 nt, standard deviation 1.15 nt).

The result that a subgroup of the 8 nt SD motifs has the ideal distance to the start codon of downstream genes prompted an analysis whether this might also be true for shorter SD motifs. First, the distances of the class of genes with less than 10 nt to the preceding gene were tabulated. Table 1 shows that the by far most populated organization of operons is a 4 nt overlap of genes (44% of all distal genes). In summary, 720 of the 792 distal genes in operons (91%) have a distance to the upstream gene less 4 nt, which means that the SD motif preceding the distal gene is localized within the open reading frame of the upstream gene. Notably, the extended SD motif GGAGGUGA contains the stop codon UGA. To enable translation of the upstream gene up to its native stop codon, the SD motif must either be localized within the ORF out of frame, or it cannot contain the UGA motif.

**Table 1:** Distances between genes in proposed operons.

Distance	No. genes
>8 nt overlap	41
8 nt overlap	57
7 nt overlap	5
4 nt overlap	352
1 nt overlap	91
0	34
1	60
2	43
3	37
4	20
5	6
6	28
7	4
8	8
9	6

**Figure 6:**
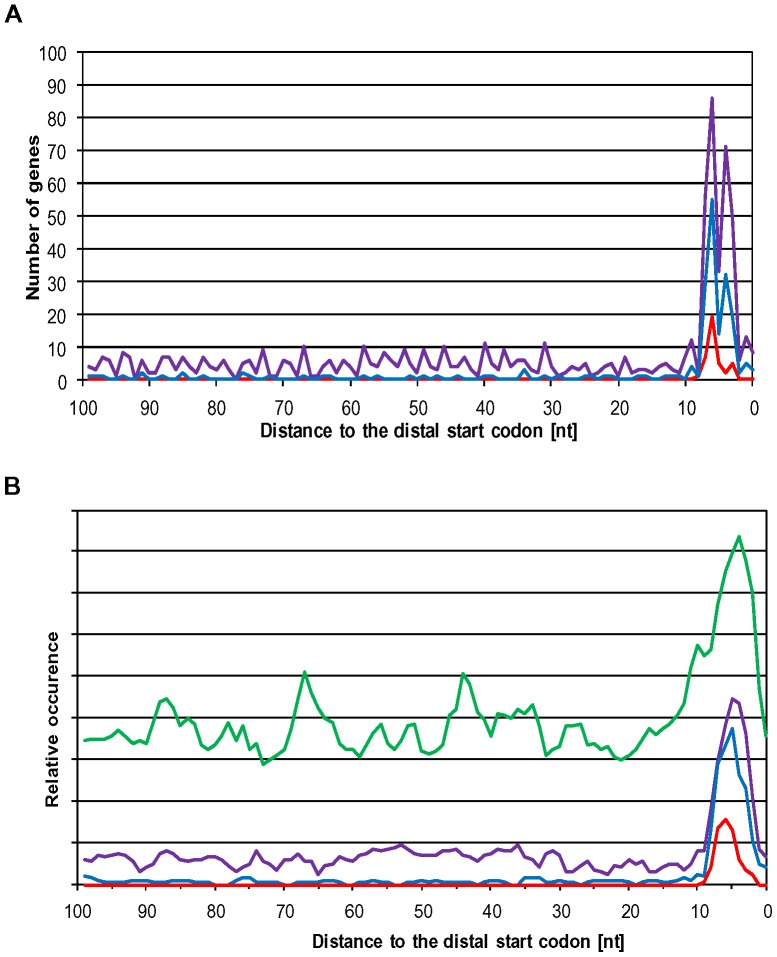
Occurrence of SD motifs upstream of distal genes in operons. 108 nt upstream of all genes with a distance of less than 10 nt to the preceding were retrieved from the genome of *H. volcanii*. The sequences were searched for the occurrence of motifs with a 5–8 nt match to the SD motif GGAGGUGA. A. the occurrence of motifs with matches of at least 6 nt (purple curve), at least 7 nt (blue curve), and 8 nt (red curve) are shown. B. smoothed curves (3 nt average) are shown for the occurrence of exactly 5 nt (green curve), exactly 6 nt (purple curve), exactly 7 nt (blue curve), and exactly 8 nt (red curve).

For all distal genes 100 nt upstream of the start codon were extracted and searched for the presence of SD motifs of varying length. Fig. 6A shows that SD motifs of at least 6 nucleotides (purple curve,  =  the 8 nt motif with up to two mismatches), at least 7 nucleotides (blue curve,  =  the 8 nt motif with up to one mismatch) and of 8 nucleotides (red curve) are present in the ideal distance of 4–6 nucleotides to the start codon, but not at any other distance. The extended 8 nt SD motif is exclusively found at a distance of 6 nt, the two slightly shorter SD motifs have two peaks at distances of 4 nt and 6 nt, while the 5 nt distance is less populated. The distance of 5 nt is probably less populated because the highest fraction of distal genes overlap with the preceding gene by 4 nt, which would be impossible if an extended SD motif including a UGA stop codon would have a distance of 5 nt to the start codon of the downstream gene (GG.AGG.UGA.NNN.NN**A.UGA**). Fig. 6B shows smoothed versions (3 nt averages) of curves of the occurrences of SD motifs of exactly 5 nt (green curve), exactly 6 nt (purple curve), exactly 7 nt (blue curve) and exactly 8 nt (red curve). It is obvious that in the GC-rich genome of *H. volcanii* “SD motifs” of 5 nt are common throughout the genome and most of them are probably non-functional. Nevertheless, there is an about twofold enrichment around a distance of 5 nt. However, SD motifs of 6 nt are very rare and SD motifs of 7–8 nt hardly occur outside of the distance range of 3–7 nt. Therefore, 6–8 nt SD motifs are highly enriched in the ideal distance for translational re-initiation at the distal start codon, strongly indicating that they are functional *in vivo*.

To analyze whether the SD motif might be functional at further sites within open reading frames in *H. volcanii*, the number of occurrences of motifs containing at least seven of the eight nucleotides of the extended GGAGGTGA motif ( =  8 nt with up to one mismatch) in the chromosome and pHV4 were quantified. The motif with at least 7 nt was found 4226 times, which is on average about once per gene. Taken together, these results indicate that *H. volcanii* makes intensive use of the SD motif within ORFs, but it does not use it for translation initiation in 5′-UTRs. Possible alternative roles of the SD motif are discussed below.

## Discussion

### Transcripts of *H. volcanii* are typically leaderless

The sequence Logo of the upstream regions of about 3000 monocistronic genes and proximal genes in operons did not reveal any translation related motifs, but the well-known archaeal basal promoter elements BRE, TATA box, -10 element and -1 pyrimidine. The distances of these transcription initiation elements to the translational start codon showed that the typical transcript of *H. volcanii* is leaderless. These results are in agreement with an experimental study that revealed that 42 of 62 haloarchaeal transcripts were leaderless [24]. A high fraction of leaderless transcripts has also been reported for other archaeal species, e.g. *H. salinarum, S. solfataricus* and *P. aerophilum* [22], [24], [39], [40]. However, this cannot be generalized to all archaea. High-throughput sequencing of the *Methanosarzina mazei* Gö1 transcriptome revealed that most transcripts contain long 5′-UTRs [41]. A bioinformatic analysis of 18 archaeal genomes had led to the prediction that two classes of archaea exist and that in one class the genes are preceded by SD motifs and thus typical transcripts should contain 5′-UTRs, while in the other class genes are preceded by promoter elements and typical transcripts are leaderless [42]. Methanogenic archaea were predicted to have transcripts with 5′-UTRs, while haloarchaea and Sulfolobales were predicted to have leaderless transcripts, which is in excellent agreement with subsequently obtained experimental results discussed above.

### The SD motif is not used for translation initiation in 5′-UTRs

While the presence of a SD motif in 5′-UTRs is not typical for haloarchaeal transcripts, inspection of the genome revealed that several genes are preceded by a SD-like motif in the optimal distance of 5+/−2 nt upstream of their start codons. If these genes had 5′-UTRs, their transcripts could make use of the SD mechanism of translation initiation. To clarify the importance of SD motifs for translation initiation in haloarchaea the *sod* gene was chosen, which has a 5′-UTR of 19 nt containing a SD motif of 7 nt with a distance of 6 nt to the start codon. A translational fusion was constructed with the *dhfr* reporter gene, and a mutant series was generated that systematically varied the base-pairing capability between the respective transcripts and the 3′-end of the 16S rRNA from 0 to 8 nt, and translational efficiencies were quantified. This is the first analysis of the importance of a SD motif in 5′-UTRs of haloarchaeal transcripts. One earlier report characterized an internal SD motif of a polycistronic transcript [33], but the functions of internal SD motifs might well differ from that in 5′-UTRs (see below). The SD mechanism of translation initiation is defined by base-pairing between the SD motif in the mRNA and the anti-SD motif at the 3′-end of the 16S rRNA. The conclusion that the SD mechanism does not operate in 5′-UTRs of *H. volcanii* transcripts is based on the following observations: 1) a series of 8 mutants with SD motifs from 0 nt to 7 nt in the *sod* 5′-UTR had identical translational efficiencies in cultures grown under three different conditions with very different growth rates (Fig. 2, 3), 2) replacement of the extended 7 nt native SD motif of the *sod* transcript by three unrelated sequences without base-pairing capability to the 16S rRNA resulted in an enhancement rather than the expected strong reduction of translation initiation (Fig. 4), 3) four randomly generated synthetic 5′-UTRs without a SD motif led to efficient translation of a reporter gene [24], 4) mutants of the upstream region of the *gvpH* gene with identical base-pairing capabilities with the 16S rRNA had very different translational efficiencies (33), 5) replacement of the extended 7 nt SD motif of the *gvpH* gene by an unrelated sequence resulted in a reduction of translational efficiency, but did not abolish translation [33], and 6) SD motifs in the required distance to start codons, which could be part of 5′-UTRs if the transcripts are not leaderless, are extremely rare in the genome of *H. volcanii*. For example, the value of 4 genes that are preceded by an 8 nt SD motif in the required distance is well below the statistical expectation of 20 occurrences that can be calculated based on the genome size and the frequencies of A, T and G.

The experimental result that SD motifs in 5′-UTRs are non-functional *in vivo* in *H. volcanii* is of general interest because a bioinformatic analysis has revealed that many species of archaea and bacteria exist that also have a low frequency of SD-motifs upstream of coding regions [18]. The bioinformatic prediction by Chang *et al.* [18] that haloarchaea contain 20–30% of genes preceded by a SD motif is probably a huge over-estimation, because motifs of only 4–5 nt, e.g. GAGG, were counted as SD motifs. Also in other phylogenetic groups the fraction of predicted SD-led genes was below 30%, e.g. in the archaea *Picrophilus, Nanoarchaeum* and most Crenarchaeota, and the bacteria *Rickettsia*, some *Mycoplasma*, *Planctomyces*, *Prochlorococcus*, *Synechocystis* and Bacteroidetes [18]. For *Prevotella bryantii*, a species of the Bacteroidetes, it has been experimentally shown that replacement of a native SD motif with an unrelated sequence reduced the translational efficiency by only 33%. The introduction of a SD motif into a 5′-UTR naturally devoid of a SD motif did not result in an increase of translational efficiency, but in contrary, to a decrease of 60% [43]. Thus the absence of the function of SD motifs in translation initiation in 5′-UTRs have now been experimentally shown for one archaeal and one bacterial species. Similar bioinformatic predictions for many additional prokaryotic species exist and it might well be also in some or all of these species the SD mechanism is non-functional.

Also in *E. coli* a small fraction of genes exists that are efficiently translated although they lack a SD motif in the 5′-UTR. In these cases translation initiation depends on the ribosomal protein S1 that binds to AU-rich sequences in the 5′-UTR [44], [45]. The S1 protein has even been hypothesized to be required for translation of most, if not all, *E. coli* transcripts [46]. However, the mechanism of initiation on SD-less 5′-UTRs must be different in bacteria and in haloarchaea which lack a homologue of the S1 protein.

The mutant containing the extended 8 nt SD motif differed drastically from the other eight mutants and had a tenfold reduced translational efficiency. This difference was unexpected, and therefore, several independent clones were analyzed, with identical results. The only possible explanation seems to be that the shorter versions of the SD motif are unable to interact with the aSD motif in the 16S rRNA, while the 8 nt SD motif triggers some kind of switch that enables base-pairing to the 3′-end of the 16S rRNA. This interaction could decrease the efficiency of switching from the initiation to the elongation phase of translation. Thus an 8 nt SD motif in 5′-UTRs is a translational repressor for *H. volcanii*. This interpretation is underscored by the bioinformatic genome analysis that revealed that in *H. volcanii* an 8 nt SD motif is localized in the required distance to a start codon of only 4 of about 4000 genes. These genes might even be leaderless and the SD motif in the genome might not be transcribed. Nevertheless, the large decrease in translational efficiency between the 7 nt SD motif and the 8 nt SD motif, which differ only by a single base-pairing capability with the 16S rRNA, remains surprising and there is no clearcut explanation.

It has also been reported for *E. coli* that extension of the SD motif lead to a decrease of translational efficiency, albeit the effects were smaller. Compared to a SD motif of 6 nt, a SD motif of 8 nt had a 30% reduced translational efficiency and a further extension to a base-pairing capability of 10 nt reduced the translational efficiency to only 17% [45]. For *E. coli* it has also been found that the optimal length of the SD motif depends on the growth temperature. While at 37°C a SD motif of 6 nt was more efficient than longer or shorter motifs, the optimal length at 20°C was 5 nt [47]. Shortening the base-pairing capability between SD and anti-SD motif from 10 to 8 and 6 nt enhanced the translational efficiency of a reporter gene fourfold and sixfold, respectively [45]. Taken together, an 8 nt SD motif is suboptimal for *E. coli*, a species that makes extensive use of the SD mechanism for translation initiation in 5′-UTRs.

### Frequent occurrence of SD motifs in ORFs and their putative functions

The observation that SD motifs occur within ORFs of *E. coli* and *B. subtilis* and that they act as pause sites for elongating ribosomes in both bacterial species [48], prompted us to analyze whether that might also be true for the haloarchaeon *H. volcanii*. The bioinformatic analysis revealed that SD motifs are overrepresented near the stop codons of proximal genes in operons. The analysis of the 100 nt preceding 792 distal genes in operons revealed that SD motifs of 6–8 nt are highly enriched at the ideal distance for translation initiation at the downstream start codon (Fig. 6). The absence of any positive effect on translation initiation of SD motifs of 5–7 nt length in 5′-UTRs (Fig. 2–3) indicates that the SD mechanism is not used to attract free ribosomal subunits in Haloferax. Therefore, it is tempting to speculate that SD motifs upstream of distant genes in operons also do not function in attracting free ribosomal subunits. This is in contrast to the role of SD motifs in distal genes in polycistronic transcripts in *E. coli*. We propose that in *H. volcanii* these SD motifs are exclusively pause sites for elongating ribosomes. They enhance the efficiency of re-initiation at the nearby start codon of the downstream gene. This hypothesis also predicts that translational coupling is much stronger in haloarchaea (and possibly other groups of archaea and bacteria) than in *E. coli*. Both predictions can and will be tested in future experiments.

A further indication that SD motifs might be differentially used in *H. volcanii* and *E. coli* is the differential spacing of genes in operons. In *H. volcanii* more than 90% of the distal genes had a distance of less than 4 nt (69% overlapped), which places the SD motif within the ORF of the upstream gene (Table 1). In contrast, in *E. coli* a distance of 9–15 bp is favored that places the SD motif in intergenic regions, where it cannot be functional for translational pausing, but for independent translation initiation of the downstream gene [49].

The fraction of 8 nt SD motifs that are not close to the 3′-end of genes was more than twofold higher than that close to the stop codon, and more than 4000 occurrences of the SD motif of 7 nt were found in the genome of *H. volcanii*. Both observations strongly suggest that SD motifs within open reading frames have additional roles to translational re-initiation at overlapping or closely spaced downstream genes. Possible functions that have been proposed for *E. coli* include 1) that the ribosome pause site is required for a high efficiency of attenuation, 2) that SD motifs within ORFs enhance the efficiency of programmed frameshifting, and 3) that SD motifs are pause sites close to downstream rare codons and prevent the ribosomes from dropping off the transcript during the extended time until a charged rare tRNA is accommodated at the A-site [48], [50]. Another, in our opinion more attractive hypothesis for the high fraction of SD motifs within ORFs in a species like *H. volcanii* is that pause sites for translation elongation are important for proper protein folding [48]. Pausing translation elongation would give the translated domains of the respective proteins the time to fold properly before the rest of the proteins are translated. Biological functions of SD motifs within open reading frames also explain the high conservation of the sequence of the 16S rRNA 3′-end even in species that do not make use of the SD motif in translation initiation in 5′-UTRs.

### Possible evolution of SD motif

The following arguments have led to the current view that translation initiation at leaderless transcripts is the evolutionary oldest mechanism: 1) leaderless transcripts exist in all three domains of life, they are predominant in some or most archaea and are the only class of transcripts in the lower eukaryote *Giardia lamblia*, 2) *in vitro* translation systems of all three domains of life have the capability to translate leaderless transcripts, and 3) strong indications have been presented that the undissociated 70S/80S ribosome and the charged initiator tRNA are sufficient for initiation at leaderless transcripts and thus leaderless transcripts would need a smaller set of initiation factors than other initiation mechanisms. In light of the fact that a variety of currently living species use leaderless translation initiation as the predominant mechanism for translation initiation and do not use the SD motif for translation initiation in 5′-UTRs, the evolutionary driving force to develop the SD motif for translational initiation is not clear. An alternative scenario seems to be that the SD motif evolved for one of the reasons discussed above, e.g. inhibiting translational failure at rare codons, efficient re-initiation of elongating ribosomes at downstream genes in polycistronic mRNAs, “buying” time for regulatory processes, or independent folding of protein domains. Only after the SD motif was well established as an intragenic pause signal for translation elongation it secondarily evolved its function in translation initiation in 5′-UTRs, which is currently used very intensively in some species (*Firmicutes, E. coli*), but not in others (haloarchaea, Bacteroidetes).

## Experimental Procedures

### Microorganisms, media and growth conditions

*H. volcanii* strain H26 was obtained from Thorsten Allers (University of Nottingham, UK) [51]. This strain contains a *pyrE2* deletion and is thus auxotrophic for uracil.

Cultures were grown aerobically in 2.1 M NaCl, pH 7.2 at 42°C as described with various C sources [24]. Complex medium contained 0.3% (w/v) yeast extract and 0.5% tryptone (w/v) and synthetic media contained, respectively, 0.5% (w/v) glucose or 20 mM sodium pyruvate or 40 mM sodium acetate. Variations in salt concentrations and temperatures are described in Results and Discussion.

*E. coli* XL1 Blue MRF' was purchased from Stratagene (Amsterdam, Netherlands) and was grown in SOB medium at 37°C.

### Plasmid constructions

All plasmids used in this study are listed in Table S1, the oligonucleotide sequences used for the plasmid construction are available upon request.

For point mutations or the deletion of single nucleotides ‘site-directed mutagenesis kit’ (Stratagene, Amsterdam, Netherlands) was used. To do so the parent plasmid was cut with restriction enzymes *Xho*I and *Kpn*I. The generated fragment contained the *dhfr* reporter gene, the 5′-UTR and the first 30 codons of the ORF region. The fragment was cloned into the shuttle vector pSKII+ (Stratagene, Amsterdam, Netherlands), where the mutagenesis was performed using specific primers (sequences upon request). After mutagenesis the fragment was cut with *Xho*I and *Kpn*I and cloned into the shuttle vector pSD1-R1/6.

For the construction of the 52 nt long extension of the 19 nt long 5′-UTR inverse PCR was used [52]. The 51 nt came from the glucose dehydrogenase of *Haloferax mediterranei.* The complete double-stranded plasmid DNA was amplified by PCR, whereas the desired mutation and the restriction sites of *Ehe*I are located in the overhanging branches. After purification, 1 µg of the PCR product was digested with *Ehe*I and ligated using T4 DNA ligase. The ligation product was incubated with *Dpn*I to digest the parental methylated DNA and then transformed into *E. coli*.

### Determination of transcript levels

RNA was isolated from exponentially growing cells as described by Chomczynski and Sacchi [53]. From these RNA preparations, 1 µg was separated on a 1.6% agarose gel and transferred to nylon membranes. A DNA-oligonucleotide probe complementary to the *dhfr* genes was used as probe for hybridization. After detection the film were scanned and the bands on the picture were analyzed with the software ‘ImageJ’ (http://rsbweb.nih.gov/ij). The background was determined locally for each band and subtracted from the measured signal. Averages and standard deviations were calculated from three independent experiments.

### Determination of protein levels and translational efficiencies

For the preparation of cell extracts for immunoblotting, 15 ml of exponentially growing cells (4*10^8^–5*10^8^ cells ml^−1^) were harvested by centrifugation (3220×g, 20 min). The pellet was suspended in 1 ml distilled water for cell lysis. Proteins were isolated as described by Wessel and Flügge [54] and dissolved in 25 mM Tris-HCl (pH 8.6). The protein concentrations were determined with the BCA protein assay reagent. Solutions of bovine serum albumin were used as standard. 70 µg of protein were incubated with 4× SDS sample buffer for 5 min at 95°C and loaded on a 15% sodium dodecyl sulphate (SDS)-polyacrylamide gel. The proteins were transferred onto a nitrocellulose membrane (Protran BA 83; Whatman, Schleicher and Schüll) by semi-dry blotting. The blots were probed with a specific DHFR antibody diluted 1:4000 in blocking solution with 0.1% Tween20. As second antibody horseradish peroxidase-conjugated goat anti-rabbit antibody (Sigma) was used at concentrations recommended by the manufacturer. Immunoreactive bands were visualized by chemiluminescence (ECL substrate). For quantification the blots from three independent experiments were scanned and pictures were analyzed with ‘ImageJ’ as described above. The translational efficiencies were calculated by dividing the protein levels by the transcript levels.

### *H. volcanii in silico* analysis

For a genome wide in silico analysis the 5′ regions of all protein coding genes of the Haloferax volcanii genome [34] and their genome position were extracted from the “Halolex” database (https://www.halolex.mpg.de) [57]. The genes were subdivided into specific groups using Microsoft Excel. For the first group all monocistronic or proximal genes in operons with an intergenic distance above 40 bp up to 150 nt were retrieved. This distance was chosen to cover both, the promoter elements and the terminator sequence of the adjacent gene. The other groups included distal genes in operons with an intergenic distance of 26–40 nt, 10–25 nt and less than 10 nt to the adjacent gene. Sequence logos were generated using RNA structure logo [55], [56].

To figure out the fraction of SD sequences in open reading frames the *H. volcanii* H26 genome sequence was scanned for SD sequences with 8 matches “GGAGGUGA” as well as one mismatch at every position. The genome localization, the distance to start or stop codons and the direction were summarized in different tables.

## Supporting Information

Table S1 Plasmids used in this study and their characteristic features.
(DOC)

Table S2 Detailed analysis of translation efficiencies of clones pPK10 – pPK18 under standard conditions (one typical experiment and normalized averages are shown in Fig. 2).
(DOC)

Table S3 Detailed analysis of translation efficiencies of clones pPK10 – pPK18 at 30°C (one typical experiment and normalized averages are shown in Fig. 3 A and B).
(DOC)

Table S4 Detailed analysis of translation efficiencies of clones pPK10 – pPK18 at acetate (one typical experiment and normalized averages are shown in Fig. 3 C and D).
(DOC)

Table S5 Detailed analysis of translation efficiencies of clones pPK19 – pPK22 under standard conditions (one typical experiment and normalized averages are shown in Fig. 4).
(DOC)
