# Supplementary material for: Haloferax volcanii, a Prokaryotic Species that Does Not Use the Shine Dalgarno Mechanism for Translation Initiation at 5′-UTRs
Source: PLoS One. 2014 Apr 14;9(4):e94979. doi: 10.1371/journal.pone.0094979 (PMC3986360; doi:10.1371/journal.pone.0094979)
Supplement: Table S1 — Plasmids used in this study and their characteristic features. (DOC) [file pone.0094979.s001.doc]

**Table S1: Plasmids used in this study and their characteristic features.**

| **Plamids** | **Features** | **Reference** |
| --- | --- | --- |
| **pSD1-R1/6** | Shuttle vector: *hdr*A under control of PrR16 promoter, selection markers Nov® und Amp® | (42) |
| **pOH16** | pMB1 derivate: 5’ UTR with of *sod2* gene with SD sequence and first 90 nt of *sod2* ORF cloned in front of *dhfr* start codon, leaderless start site AUG at the 5’-end of the 5’-UTR | This study |
| **pPK10** | pOH16 derivate without artificial leaderless start site and mutation of SD sequence from GGAGGUUA to AACAAAAC | This study |
| **pPK11** | pPK10 derivate with mutated SD sequence from AACAAAAC to AACAAAAA | This study |
| **pPK12** | pPK10 derivate with mutated SD sequence from AACAAAAC to AACAAAGA | This study |
| **pPK13** | pPK10 derivate with mutated SD sequence from AACAAAAC to AACAAUGA | This study |
| **pPK14** | pPK10 derivate with mutated SD sequence from AACAAAAC to AACAGUGA | This study |
| **pPK15** | pPK10 derivate with mutated SD sequence from AACAAAAC to AACGGUGA | This study |
| **pPK16** | pPK10 derivate with mutated SD sequence from AACAAAAC to AAAGGUGA | This study |
| **pPK17** | pPK10 derivate with mutated SD sequence from AACAAAAC to AGAGGUGA | This study |
| **pPK18** | pPK10 derivate with mutated SD sequence from AACAAAAC to GGAGGUGA | This study |
| **pPK19** | pOH16 derivate with an insertion of 51 nt of the *gdh* ORF into the 5‘-UTR of *sod*2 gene upstream of the SD sequence | This study |
| **pPK20** | pPK19 derivate with mutated SD sequence from GGAGGUUA to CCUCCACC | This study |
| **pPK21** | pPK19 derivate with mutated SD sequence from GGAGGUUA to ACGUCCUG | This study |
| **pPK22** | pPK19 derivate with mutated SD sequence from GGAGGUUA to UCCUCGAC | This study |
