# Supplementary material for: Haloferax volcanii, a Prokaryotic Species that Does Not Use the Shine Dalgarno Mechanism for Translation Initiation at 5′-UTRs
Source: PLoS One. 2014 Apr 14;9(4):e94979. doi: 10.1371/journal.pone.0094979 (PMC3986360; doi:10.1371/journal.pone.0094979)
Supplement: Table S2 — Detailed analysis of translation efficiencies of clones pPK10 – pPK18 under standard conditions (one typical experiment and normalized averages are shown in Fig. 2). (DOC) [file pone.0094979.s002.doc]

Table S2. Detailed analysis of translation efficiencies of clones pPK10 – pPK18 under standard conditions (one typical experiment and normalized averages are shown in Fig. 2)

|  | **10** | **11** | **12** | **13** | **14** | **15** | **16** | **17** | **18** |
| --- | --- | --- | --- | --- | --- | --- | --- | --- | --- |
| **Protein level (relative units)** | 1,14 (0,17) | 1,22 (0,09) | 1,04 (0,05) | 1,09 (0,16) | 1,40 (0,18) | 1,11 (0,20) | 0,98 (0,11) | 0,94 (0,24) | 0,07 (0,04) |
| **Transcript level (relative units)** | 1,15 (0,17) | 1,18 (0,25) | 1,26 (0,24) | 1,05 (0,27) | 1,17 (0,29) | 0,81 (0,13) | 0,93 (0,25) | 0,97 (0,03) | 0,80 (0,16) |
| **Translation efficiency (relative units)** | 1,00 (0,08) | 1,06 (0,14) | 0,86 (0,14) | 1,14 (0,47) | 1,31 (0,44) | 1,40 (0,28) | 1,16 (0,40) | 0,97 (0,25) | 0,09 (0,07) |
