# Supplementary material for: Haloferax volcanii, a Prokaryotic Species that Does Not Use the Shine Dalgarno Mechanism for Translation Initiation at 5′-UTRs
Source: PLoS One. 2014 Apr 14;9(4):e94979. doi: 10.1371/journal.pone.0094979 (PMC3986360; doi:10.1371/journal.pone.0094979)
Supplement: Table S3 — Detailed analysis of translation efficiencies of clones pPK10 – pPK18 at 30°C (one typical experiment and normalized averages are shown in Fig. 3 A and B). (DOC) [file pone.0094979.s003.doc]

Table S3. Detailed analysis of translation efficiencies of clones pPK10 – pPK18 at 30^o^C (one typical experiment and normalized averages are shown in Fig. 3 A and B).

|  | **10** | **11** | **12** | **13** | **14** | **15** | **16** | **17** | **18** |
| --- | --- | --- | --- | --- | --- | --- | --- | --- | --- |
| **Protein level (relative units)** | 0,89 (0,14) | 1,28 (0,28) | 1,08 (0,06) | 1,00 (0,26) | 1,06 (0,07) | 0,97 (0,12) | 1,19 (0,06) | 1,20 (0,08) | 0,33 (0,05) |
| **Transcript level (relative units)** | 1,04 (0,20) | 0,90 (0,07) | 1,05 (0,31) | 1,02 (0,14) | 1,12 (0,04) | 1,03 (0,22) | 0,95 (0,24) | 1,01 (0,09) | 1,05 (0,13) |
| **Translation efficiency (relative units)** | 0,87 (0,08) | 1,41 (0,20) | 1,15 (0,41) | 1,03 (0,37) | 0,95 (0,09) | 0,97 (0,11) | 1,37 (0,45) | 1,20 (0,19) | 0,32 (0,07) |
