# Supplementary material for: Haloferax volcanii, a Prokaryotic Species that Does Not Use the Shine Dalgarno Mechanism for Translation Initiation at 5′-UTRs
Source: PLoS One. 2014 Apr 14;9(4):e94979. doi: 10.1371/journal.pone.0094979 (PMC3986360; doi:10.1371/journal.pone.0094979)
Supplement: Table S4 — Detailed analysis of translation efficiencies of clones pPK10 – pPK18 at acetate (one typical experiment and normalized averages are shown in Fig. 3 C and D). (DOC) [file pone.0094979.s004.doc]

Table S4. Detailed analysis of translation efficiencies of clones pPK10 – pPK18 at acetate (one typical experiment and normalized averages are shown in Fig. 3 C and D).

|  | **10** | **11** | **12** | **13** | **14** | **15** | **16** | **17** | **18** |
| --- | --- | --- | --- | --- | --- | --- | --- | --- | --- |
| **Protein level (relative units)** | 1,39 (0,29) | 0,96 (0,16) | 1,21 (0,17) | 1,11 (0,30) | 0,81 (0,25) | 1,08 (0,29) | 1,08 (0,28) | 1,17 (0,17) | 0,20 (0,00) |
| **Transcript level (relative units)** | 145 (0,26) | 1,12 (0,20) | 1,13 (0,20) | 1,22 (0,11) | 0,90 (0,16) | 0,92 (0,06) | 0,86 (0,08) | 0,83 (0,16) | 0,80 (0,13) |
| **Translation efficiency (relative units)** | 1,02 (0,37) | 0,81 (0,30) | 1,08 (0,04) | 0,90 (0,18) | 0,92 (0,34) | 1,16 (0,25) | 1,23 (0,22) | 1,52 (0,52) | 0,26 (0,05) |
