# Supplementary material for: Haloferax volcanii, a Prokaryotic Species that Does Not Use the Shine Dalgarno Mechanism for Translation Initiation at 5′-UTRs
Source: PLoS One. 2014 Apr 14;9(4):e94979. doi: 10.1371/journal.pone.0094979 (PMC3986360; doi:10.1371/journal.pone.0094979)
Supplement: Table S5 — Detailed analysis of translation efficiencies of clones pPK19 – pPK22 under standard conditions (one typical experiment and normalized averages are shown in Fig. 4). (DOC) [file pone.0094979.s005.doc]

Table S5. Detailed analysis of translation efficiencies of clones pPK19 – pPK22 under standard conditions (one typical experiment and normalized averages are shown in Fig. 4).

|  |  | **19** | **20** | **21** | **22** |
| --- | --- | --- | --- | --- | --- |
| **Protein level (relative units)** | **1^st^  AUG** | 0,39 (0,54) | 0,10 (0,08) | 0,00 (0,04) | 0,00 (0,03) |
|  | **2^nd^ AUG** | 1,03 (0,67) | 1,44 (0,06) | 0,85 (0,09) | 1,00 (0,17) |
| **Transcript level (relative units)** |  | 1,45 (0,25) | 0,81 (0,12) | 0,83 (0,16) | 0,89 (0,16) |
| **Translation efficiency (relative units)** | **1^st^  AUG** | 0,23 (0,32) | 0,13 (011) | 0,00 (0,05) | 0,00 (0,03) |
|  | **2^nd^ AUG** | 0,65 (0,37) | 1,81 (0,24) | 1,05 (0,12) | 1,13 (0,15) |
